# AI anxiety and teacher innovative intentions: a moderated serial mediation model of burnout and creative self-efficacy

**DOI:** 10.3389/fpsyg.2026.1828689

**Published:** 2026-05-28

**Authors:** Tongqiang Dong, Ziyi Yang, Yu Fang, Jianying Che, Lanxin Li, Yong Kong

**Affiliations:** School of Communication, Qufu Normal University, Rizhao, China

**Keywords:** AI anxiety, conservation of resources theory, creative self-efficacy, job burnout, social cognitive theory, teacher innovation

## Abstract

**Introduction:**

The proliferation of generative artificial intelligence (AI) in education presents a double-edged sword: while promising transformative pedagogical potential, it simultaneously triggers significant “AI anxiety” among educators. Despite its relevance, the psychological mechanisms through which this specific anxiety impedes teachers’ willingness to innovate remain underexplored.

**Methods:**

Based on Conservation of Resources (COR) theory and Social Cognitive Theory (SCT), this study constructs a moderated serial mediation model. Data were collected from 528 K-12 teachers via a stratified random sampling method. Structural equation modeling (SEM) and bootstrapping techniques were employed to test the hypotheses.

**Results:**

Findings indicate that (1) AI anxiety positively predicts job burnout; (2) Job burnout and creative self-efficacy serially mediate the relationship between AI anxiety and innovative intentions (AI Anxiety → Burnout → Lower Creative Self-Efficacy → Reduced Innovative Intentions); and (3) Perceived Organizational Support (POS) acts as a critical moderator, buffering the deleterious effect of AI anxiety on burnout and attenuating the indirect negative effect on innovative intentions.

**Discussion:**

Moving beyond mere variable relationships, this study reveals a tangible process-based mechanism of teacher resistance to AI: the “depletion → cognition → behavior” pathway. It highlights that anxiety suppresses innovation not merely through emotional exhaustion, but by systematically translating resource depletion into a cognitive deficit (eroded self-efficacy), which ultimately drives behavioral withdrawal.

## 1 Introduction

The integration of artificial intelligence (AI) into educational ecosystems has accelerated at an unprecedented pace, particularly with the advent of generative models such as ChatGPT and other Large Language Models (LLMs). This technological disruption has fundamentally altered the educational landscape, precipitating a complex, dualistic scenario for educators worldwide. On one hand, AI offers transformative opportunities to revolutionize pedagogy, enabling hyper-personalized learning pathways and significant administrative efficiencies (Chen et al., 2020; Chiu, 2024; Hwang et al., 2020). On the other hand, the ubiquity of these intelligent systems introduces a profound existential threat. Fearing not just a change in tools but a potential replacement of the self, educators are increasingly experiencing a unique psychological burden conceptualized as “AI anxiety.”

Despite the growing recognition of this phenomenon, a fundamental question remains: How exactly does this specific anxiety erode a teacher’s willingness to innovate? While routine technology-related stress is known to impact general job satisfaction, educational innovation is a unique, high-stakes cognitive endeavor requiring significant emotional and cognitive investment. To unravel this complexity, this study proposes a cohesive framework by integrating Conservation of Resources (COR) theory with Social Cognitive Theory (SCT). We aim to map the complete psychological trajectory from the external stressor (AI anxiety) to behavioral withdrawal (reduced innovative intentions). Specifically, we investigate the serial mediating roles of job burnout and creative self-efficacy, alongside the protective moderating role of Perceived Organizational Support (POS).

In doing so, this study makes three significant contributions to the literature on educational technology and organizational psychology. First, it moves beyond the prevailing “pro-innovation bias” to empirically capture the psychological costs of AI integration, highlighting AI anxiety as a critical drain on teachers’ resources. Second, it advances theoretical innovation by providing a process-based explanation for teacher resistance. Moving beyond mere variable relationships, it maps a tangible “depletion → cognition → behavior” mechanism, clarifying that anxiety suppresses innovation not merely through emotional exhaustion, but by systematically eroding professional self-efficacy. Third, by establishing organizational support as a pivotal boundary condition, this research provides actionable insights for educational institutions to foster a psychologically safe “innovation sandbox” in the AI era.

## 2 Literature review

### 2.1 The shift from technostress to AI anxiety in education

The integration of technology in education has long been associated with technostress–the negative psychological link between people and the introduction of new technologies (Ragu-Nathan et al., 2008). Historically, technostress has been conceptualized around operational frustrations, such as hardware failures, software usability issues, or the pressure to remain constantly connected. However, the emergence of generative AI (e.g., ChatGPT) has necessitated a paradigm shift in educational technology literature. Scholars argue that AI introduces a distinct psychological burden conceptualized as “AI anxiety” (Wang and Wang, 2022). Unlike traditional tools, AI encompasses deeper existential apprehensions regarding the opacity of algorithmic decision-making and the looming potential for professional displacement. It threatens the teacher’s core professional identity as the primary “knower” in the classroom (Pan et al., 2025). Consequently, recent literature emphasizes that AI anxiety must be examined as a unique stressor, distinct from routine technological fatigue. However, the empirical literature on AI anxiety in K-12 teaching contexts remains notably limited. Extant studies have predominantly sampled university students or knowledge workers in corporate settings, leaving the specific psychological dynamics of schoolteachers–whose professional identity is anchored in relational authority and irreplaceable pedagogical judgment–largely unexamined. Furthermore, the measurement of AI anxiety has lacked consistency across studies: some operationalize it as a unidimensional technophobia construct, while others decompose it into cognitive appraisal and somatic arousal components (Wang and Wang, 2022), a conceptual inconsistency that complicates cross-study comparisons and undermines cumulative knowledge-building.

### 2.2 Psychological drivers of teacher innovation

In the face of technological disruption, pedagogical innovation is widely advocated. However, the literature on organizational psychology indicates that innovation is not an automatic response to new tools; it is a high-stakes, resource-intensive cognitive endeavor. According to Bandura (1997) and recent educational research (Thurlings et al., 2015), teachers’ innovative behaviors are fundamentally driven by “agentic beliefs,” specifically creative self-efficacy (CSE). Furthermore, engaging in innovative practices requires a robust reserve of “energetic resources” (Hobfoll, 1989). When educators are depleted of emotional vitality, their capacity to formulate novel solutions is severely compromised. Existing studies have documented the negative relationship between general stress and job performance (Bakker and Demerouti, 2017), but how specific emotional depletion impacts the cognitive prerequisites for pedagogical innovation remains a complex issue requiring further theoretical integration. A closer inspection of the relevant literature reveals that most studies address only one node of this psychological chain. Research grounded in COR theory tends to terminate at burnout as the outcome variable, documenting emotional exhaustion without tracing its downstream cognitive consequences (Salmela-Aro et al., 2019; Skaalvik and Skaalvik, 2017). Conversely, SCT-informed studies often treat self-efficacy as an antecedent to innovation while assuming stable emotional baselines, thus neglecting the resource-depletion processes that precede efficacy appraisal (Gong et al., 2009; Urban and Urban, 2025). This bifurcation leaves a theoretical lacuna: precisely how emotional exhaustion is cognitively transformed into a belief of incapacity–and how that belief, rather than exhaustion itself, ultimately suppresses behavioral intention–remains underspecified.

### 2.3 Current literature gaps and the present study

While the existing literature provides a robust foundation for understanding AI anxiety and teacher innovation independently, a critical synthesis reveals two major research gaps. First, the prevailing EdTech literature has systematically underweighted the psychological costs of AI integration. The dominant theoretical lens–the Technology Acceptance Model (TAM) and its derivatives–was developed in the context of routine workplace software and implicitly assumes that technological exposure generates positive adoption intentions when perceived usefulness and ease of use are sufficiently high (Davis, 1989). Recent studies applying TAM or its extensions to generative AI (Chiu, 2024; Hwang et al., 2020) have preserved this optimistic framing, often treating anxiety as a minor moderator rather than a structurally significant mechanism. What these accounts omit is the qualitatively distinct threat that generative AI poses to teachers’ professional self-concept–a threat that cannot be accommodated within a utility-calculus model and that COR theory is better positioned to explain. Second, even studies that acknowledge the psychological costs of AI integration rarely trace the sequential mechanism through which anxiety translates into behavioral withdrawal–the precise “depletion → cognition → behavior” chain remains empirically unverified in the K-12 teacher context.”

To bridge these gaps, the present study moves beyond establishing mere variable relationships to investigate the process-based mechanism underlying teacher resistance. Specifically, we explore how AI anxiety impedes innovative intentions by tracing the “depletion → cognition → behavior” pathway, thereby providing a much-needed analytical clarity to the current literature.

## 3 Theoretical models and hypotheses

### 3.1 Theoretical framework: synergizing COR and SCT

To comprehensively delineate the psychological mechanisms linking AI anxiety to teacher innovative intentions, this study integrates Conservation of Resources (COR) theory (Hobfoll, 1989; Halbesleben et al., 2014) with Social Cognitive Theory (SCT) (Bandura, 1986). While these theories are distinct, their synthesis offers a robust lens for understanding the “stress-to-withdrawal” trajectory. Independent application of either theory would yield an incomplete picture: COR primarily addresses the emotional and physiological depletion caused by stressors but is less precise in explaining how this depletion specifically inhibits complex cognitive tasks like innovation. Conversely, SCT explains human agency and efficacy beliefs but often treats physiological states as background factors rather than central drivers.

By bridging these frameworks, we construct a “depletion-to-cognition” model. It is necessary to distinguish this synthesis from superficially similar dual-process frameworks in the organizational stress literature. Several prior studies have combined emotional depletion and self-efficacy in mediation analyses; however, they typically specify these variables as parallel mediators operating independently along separate paths (Bakker and Demerouti, 2017; Liu et al., 2016). Such parallel specifications assume that burnout and self-efficacy are both direct downstream products of the stressor, implying no causal dependency between them. The present model departs from this convention by theorizing a sequential dependency: burnout is not merely co-present with diminished self-efficacy but is its proximal cognitive antecedent. This serial architecture is grounded in Bandura’s (1997) explicit claim that physiological and affective states constitute a primary informational input through which individuals form efficacy judgments–a claim that requires emotional depletion to be causally prior to efficacy appraisal, not merely correlated with it. In specifying this directionality, the present study moves beyond variable-level association toward a mechanism-level explanation of how stress becomes inaction. COR theory serves as the overarching framework for understanding the stress process. Its central tenet is that individuals are motivated to obtain, retain, and protect resources–defined as objects, conditions, personal characteristics, or energies that are valued. Stress arises when these resources are threatened or lost. In the context of AI integration, anxiety represents a perceived threat to professional stability and competence, triggering a defensive mode of resource conservation. Complementing this, SCT illuminates the specific cognitive pathways through which resource depletion translates into behavioral inaction. SCT asserts that human agency is governed by self-regulatory mechanisms, with self-efficacy serving as the pivotal driver. We argue that the physiological and emotional exhaustion predicted by COR constitutes the somatic input that teachers use to formulate their cognitive appraisals of incompetence, as described by SCT.

### 3.2 AI anxiety and job burnout: the resource depletion path

According to the primacy of resource loss principle in COR theory, resource loss is disproportionately more salient and impactful than resource gain. The advent of generative AI acts not as a discrete event but as a chronic environmental stressor, threatening teachers’ key resources such as job security, professional identity, and perceived control over the classroom ecosystem. Unlike traditional tools, AI is autonomous and opaque; coping with this persistent ambiguity requires a continuous investment of cognitive and emotional effort–for instance, constantly monitoring technological updates, verifying AI-generated content for hallucinations, or ruminating on future obsolescence.

This sustained compensatory effort drains the individual’s reservoir of psychological resources. In the COR framework, when individuals invest resources (time, effort, attention) without receiving a corresponding return (reduced threat, mastery), they experience a “loss spiral.” In the case of AI anxiety, the threat is often amorphous and evolving, meaning the teacher’s efforts rarely yield a sense of complete safety. Consequently, the individual enters a state of resource bankruptcy. This state manifests as job burnout, specifically the dimension of emotional exhaustion, which represents the endpoint of a resource loss spiral where the teacher lacks the energy to meet psychological work demands (Maslach et al., 2001; Bakker and Demerouti, 2017; Salmela-Aro et al., 2019). Thus, AI anxiety functions as a mechanism of resource erosion, converting professional uncertainty into emotional depletion.

Hypothesis 1 (H1): AI anxiety is positively related to job burnout.

### 3.3 The serial mediation: from exhaustion to doubt to withdrawal

To elucidate how burnout suppresses innovation, we must explicate the transition from an emotional state to a cognitive deficit. Innovation is an inherently demanding and risky activity that requires high levels of creative self-efficacy (CSE)–the specific confidence in one’s ability to generate novel and useful ideas (Tierney and Farmer, 2002; Gong et al., 2009; Urban and Urban, 2025). It is not enough to have the skill to innovate; one must have the belief in that skill to deploy it under uncertainty.

Bandura (1997) identified physiological and affective states as one of the four principal sources of self-efficacy information. Individuals act as interpreters of their own somatic feedback; high arousal, fatigue, and exhaustion are typically interpreted as signs of vulnerability and incompetence. Therefore, a teacher suffering from burnout (a resource-depleted state per COR) is unlikely to attribute their fatigue to external workload alone. Instead, they are prone to negatively interpreting their exhaustion as an internal lack of capability (a cognitive appraisal per SCT). “I am too tired to deal with this” easily morphs into “I am not capable of dealing with this.” This negative physiological feedback significantly erodes their creative self-efficacy.

Subsequently, within the SCT framework, self-efficacy is the proximal determinant of behavioral choice, particularly regarding how much effort people will expend and how long they will persist in the face of obstacles. A critical theoretical distinction must be made here regarding the behavioral response to AI. While some stress theories might suggest a “compensatory effort” (i.e., working harder to overcome the threat), SCT posits that when efficacy beliefs drop below a critical threshold, the dominant response shifts to “avoidance behavior” (Bandura, 1997; Liu et al., 2016). This is especially pertinent in the context of AI integration, which represents a high-uncertainty, high-stakes environment. Teachers with diminished CSE perceive the task of innovating with AI not as a manageable challenge, but as a threat that exceeds their coping capabilities. To protect their remaining self-esteem and prevent further resource loss, they strategically withdraw from the innovative task. Consequently, the erosion of self-efficacy acts as a cognitive gatekeeper that blocks the translation of potential ability into intentional action.

This logic establishes a sequential “domino effect”: AI anxiety drains emotional vitality (burnout), which in turn undermines cognitive confidence (low CSE), ultimately leading to behavioral withdrawal. Specifically, consistent with SCT, teachers operate as agentic decision-makers who weigh their internal resources against task demands. When burnout signals a critical depletion of energetic resources (the COR perspective), teachers cognitively appraise this state as an inability to meet the high demands of creativity (the SCT perspective). Thus, the physiological state of exhaustion is translated into the cognitive state of doubt (low CSE), creating a bridge between resource loss and behavioral withdrawal.

Hypothesis 2 (H2): Job burnout and creative self-efficacy serially mediate the relationship between AI anxiety and teacher innovative intentions (AI Anxiety → Job Burnout → Creative Self-Efficacy → Innovative Intentions).

### 3.4 The moderating role of Perceived Organizational Support (POS)

While AI anxiety initiates a potential loss spiral, the organizational context can significantly alter this trajectory. Perceived Organizational Support (POS)–the extent to which the school values teachers’ contributions and cares for their well-being–serves as a critical contextual resource in the COR framework (Eisenberger et al., 1986; Kurtessis et al., 2017).

We posit that POS functions as a “resource buffer” at the initial stage of the stress process. When teachers perceive high levels of organizational support, the threat posed by AI is mitigated by the assurance of institutional backing (technical training, emotional reassurance, and tolerance for failure). This injection of external resources compensates for the internal resources consumed by anxiety. It alters the teacher’s cognitive appraisal of the situation: the AI challenge becomes manageable rather than overwhelming. Conversely, in a low-support environment, teachers must cope with the AI threat in isolation, accelerating the depletion of their emotional resources and hastening the onset of burnout.

Hypothesis 3 (H3): Perceived Organizational Support moderates the relationship between AI anxiety and job burnout, such that the positive relationship is weaker when POS is high.

### 3.5 A moderated serial mediation model

Integrating the serial mediation logic with the buffering effect of POS, we propose a moderated serial mediation framework. Specifically, this study posits a “first-stage moderation” model (Hayes, 2017), where the organizational context determines the strength of the initial stress reaction. If organizational support successfully attenuates the link between AI anxiety and burnout (as proposed in H3), it effectively blocks the pathogenic chain at its inception. By keeping burnout levels in check through high POS, the subsequent erosion of creative self-efficacy is prevented, and the downstream effect on innovative intentions is minimized. In essence, POS acts as a protective gatekeeper; high support weakens the transmission of anxiety into the exhaustion-doubt-withdrawal trajectory.

**FIGURE 1:**
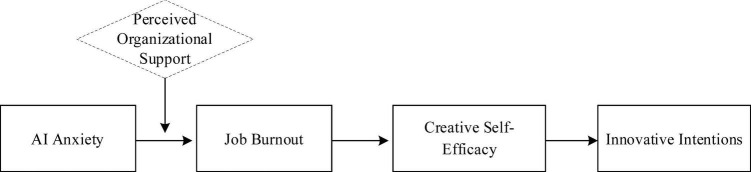
Conceptual model.

Hypothesis 4 (H4): Perceived Organizational Support moderates the serial indirect effect of AI anxiety on teacher innovative intentions via job burnout and creative self-efficacy, such that this negative indirect effect is weaker (attenuated) when POS is high. The proposed theoretical model is illustrated in Figure 1.

## 4 Materials and methods

This section delineates the methodological framework employed to empirically test the proposed moderated serial mediation model. It details the research design, sampling procedures, measurement instrumentation, and the analytical strategy adopted to ensure the robustness of the findings.

### 4.1 Participants and procedure

To test the hypotheses, a cross-sectional survey design was utilized. Data were collected from in-service K-12 teachers across Eastern China during the Fall 2025 academic semester. We employed a stratified random sampling technique, stratifying by school level (primary, middle, and high school) to ensure the sample accurately reflected the demographic composition of the regional teaching workforce. The target sample size was determined *a priori* using G*Power 3.1 software. Based on a medium effect size (f^2^ = 0.15), an alpha level of 0.05, and a power of 0.95 with five predictors, the minimum required sample size was calculated to be 138. To account for potential invalid responses and to ensure sufficient power for complex structural equation modeling (SEM), we aimed to collect at least 500 valid responses, satisfying the recommendation of N > 200 for SEM analysis (Kline, 2015).

The survey was administered via a secure online platform. Given that all variables were self-reported, we implemented rigorous procedural remedies to mitigate potential Common Method Bias (CMB) at the design stage (Podsakoff et al., 2003). First, we employed a psychological separation strategy to reduce artifactual covariation. Specifically, the independent variable (AI anxiety) was placed at the beginning of the survey, while the dependent variable (innovative intentions) was positioned at the very end. These key measures were separated by filler items and demographic questions to disrupt the respondents’ ability to infer cause-and-effect relationships. Second, item ambiguity was reduced through a pilot test, and the order of questions was counterbalanced. Third, to minimize social desirability bias, anonymity was strictly assured. Participants were informed that their responses would be encrypted and used solely for academic purposes, with no right or wrong answers.

A total of 600 questionnaires were distributed. Following data cleaning, 72 responses were excluded due to incomplete answers, logical inconsistencies, or failure to pass attention checks. This resulted in a final valid sample of 528 teachers, yielding an effective response rate of 88.0%. The demographic profile of the sample was diverse: 65.0% were female, the mean age was 34.5 years (SD = 5.2), and the average teaching tenure was 8.2 years (SD = 4.1). Additionally, 78.0% of participants held a bachelor’s degree or higher.

### 4.2 Measures

All constructs were measured using established scales with proven psychometric properties in prior literature. To ensure linguistic equivalence for the Chinese context, we followed the standard translation-back-translation procedure (Brislin, 1986). A panel of three experts in educational psychology and educational technology reviewed the translated items for content validity. Unless otherwise noted, all items were rated on a 5-point Likert scale ranging from 1 (strongly disagree) to 5 (strongly agree).

Artificial intelligence Anxiety (Independent Variable): We adapted the scale developed by Wang and Wang (2022), modifying the context to specifically address anxiety related to generative AI integration. The scale comprises 4 items (“I feel anxious about the complexity of using AI in my teaching,” “I worry that AI might replace some of my teaching functions”). The adaptation process proceeded in three stages. First, items referencing generic “technology” or “computers” were revised to explicitly reference “generative AI tools” (e.g., ChatGPT, AI-based grading systems), reflecting the qualitative shift in the nature of the technological stressor. Second, two items were modified to capture the professional-identity dimension of AI anxiety–specifically, the perceived threat of role displacement–which Wang and Wang (2022) identified as conceptually distinct from operational frustration but which required more direct expression in the K-12 teaching context. Third, the revised items were subjected to content validity review by the three-expert panel described above; all items were retained following consensus evaluation. To verify that the adapted scale retained its unidimensional structure in the present sample, a single-factor CFA was conducted prior to the full measurement model analysis. The results confirmed an acceptable fit (χ^2^/df = 1.98, CFI = 0.97, RMSEA = 0.04), providing evidence that the adapted items converged on a coherent construct. The scale demonstrated high internal consistency (Cronbach’s α = 0.89).

Job burnout (mediator 1): in line with the well-established conceptualization that emotional exhaustion represents the core dimension of burnout, this construct was measured using the Emotional Exhaustion subscale from the Chinese Occupational Burnout Scale [adapted and validated by Li and Shi (2003)]. This scale was specifically developed and psychometrically validated within the Chinese cultural context. The subscale comprises 5 items assessing the extent of emotional resource depletion (e.g., “I feel emotionally drained from my work”). All items were rated on a 7-point Likert scale (from 1 = never to 7 = every day). The Cronbach’s α for this subscale was 0.88.

Creative self-efficacy (mediator 2): to assess teachers’ confidence in their creative capabilities, we utilized the scale developed by Tierney and Farmer (2002), adapted for the educational innovation context. This 3-item scale measures the belief in one’s ability to generate novel ideas (“I have confidence in my ability to solve problems creatively”). As Tierney and Farmer (2002) developed and validated this scale in a manufacturing context, its adaptation for AI-integrated teaching required careful attention to construct alignment. Item language was revised to anchor the confidence appraisal in pedagogical creativity specifically–for instance, referencing the generation of novel instructional approaches rather than product innovation. This adaptation ensures conceptual fidelity to the core construct–perceived capacity for creative ideation–while grounding it in the behavioral domain relevant to the present study. The adapted items were reviewed by the expert panel for content validity, and the factor loadings obtained in the CFA (range: 0.79–0.86) indicate that the items converge adequately on the intended construct within this sample. The Cronbach’s α was 0.87.

Innovative intentions (dependent variable): innovative intentions were operationalized as the willingness to adopt and implement new ideas, measured using a scale adapted from Scott and Bruce (1994). The 4 items reflect a proactive orientation toward AI adoption (“I intend to search out new AI-based teaching methods/techniques”). The Cronbach’s α was 0.90.

Perceived Organizational Support (moderator): we employed a shortened version of the Survey of Perceived Organizational Support (Eisenberger et al., 1986). This 4-item scale assesses teachers’ global beliefs concerning the extent to which the school values their contributions and cares about their well-being (“The school cares about my well-being,” “Help is available from the school when I have a problem”). The Cronbach’s α was 0.88.

Control variables: consistent with previous research on teacher innovation (Thurlings et al., 2015), we controlled for demographic variables that might confound the results, including gender (dummy coded), age (in years), teaching tenure (in years), and prior experience with AI tools (1 = Yes, 0 = No).

### 4.3 Data analysis strategy

Data analysis was conducted using IBM SPSS 26.0 and AMOS 24.0. The analytical procedure followed a three-phase approach:

Preliminary analysis & measurement model: first, we conducted Confirmatory Factor Analysis (CFA) using AMOS to evaluate the distinctiveness and construct validity of the measurement model. Model fit was assessed using established indices: χ^2^/df, Comparative Fit Index (CFI), Tucker-Lewis Index (TLI), Root Mean Square Error of Approximation (RMSEA), and Standardized Root Mean Square Residual (SRMR). We also examined convergent validity [via Average Variance Extracted (AVE) and Composite Reliability (CR) and discriminant validity (via the Fornell-Larcker criterion)]. Furthermore, Harman’s single-factor test was performed to check for Common Method Bias (CMB).

Descriptive Statistics: Descriptive statistics (means, standard deviations) and Pearson correlation coefficients were calculated to examine the initial bivariate relationships among the study variables and to check for multicollinearity.

Hypothesis testing: to test the hypothesized moderated serial mediation model, we utilized the PROCESS macro for SPSS (Hayes, 2017). Specifically, Model 6 was used to test the serial mediation effect (AI Anxiety → Burnout → Creative Self-Efficacy → Innovative Intentions), and Model 83 was employed to test the moderated mediation effect. We used a bootstrapping technique with 5,000 resamples to generate bias-corrected 95% confidence intervals (CIs). An effect was considered statistically significant if the 95% CI did not contain zero.

### 4.4 Ethical considerations

This study involved the participation of 528 human subjects (K-12 teachers). All research procedures involving human participants were conducted in strict accordance with the ethical standards of the institutional research committee and with the 1964 Helsinki Declaration and its later amendments. The experimental protocol, including the survey instruments and data management plans, was formally reviewed and approved by the Institutional Review Board (IRB) of Qufu Normal University (Approval Number: 2025164).

A rigorous informed consent process was implemented prior to data collection. All participants were provided with a digital informed consent form detailing the study’s purpose, voluntary nature of participation, data confidentiality, and their right to withdraw at any time without penalty or consequence. Participants were required to explicitly click an “I Agree” button to indicate their informed consent before proceeding to the survey. To ensure privacy, all collected data were completely anonymized, and no personally identifiable information (PII) was retained.

## 5 Results

### 5.1 Measurement model assessment and common method bias

Before testing the hypotheses, we evaluated the measurement model using Confirmatory Factor Analysis (CFA). The hypothesized five-factor model (AI anxiety, job burnout, creative self-efficacy, innovative intentions, and Perceived Organizational Support) demonstrated a satisfactory fit to the data (χ^2^/df = 2.14, CFI = 0.95, TLI = 0.94, RMSEA = 0.05, SRMR = 0.04). We compared this model with alternative models, including a one-factor model where all items were loaded onto a single construct. The results indicated that the five-factor model fit the data significantly better than any alternative model (Δχ^2^ = 560.20, *p* < 0.001), confirming the distinctiveness of the study variables. To further examine discriminant validity, we additionally compared the hypothesized five-factor model against a four-factor model collapsing the two constructs with the highest inter-correlation–creative self-efficacy and innovative intentions (*r* = 0.62)–onto a single factor. The five-factor model demonstrated significantly superior fit (Δχ^2^ = 134.60, df = 4, *p* < 0.001), confirming that although these constructs are substantially related, they represent empirically distinguishable psychological entities. This distinction is theoretically consequential: self-efficacy, per SCT, refers to a cognitive belief state, while innovative intention represents a behavioral motivational orientation; conflating them would obscure the mechanism through which efficacy appraisal translates into behavioral predisposition.

Given that data were collected from a single source, we assessed Common Method Bias (CMB) using Harman’s single-factor test. The unrotated factor analysis revealed 5 factors with eigenvalues greater than 1.0, with the first factor accounting for 28.5% of the total variance. This is well below the 50% threshold, suggesting that CMB is not a pervasive issue in this study. Detailed psychometric properties of the constructs are presented in Table 1.

**TABLE 1:** Reliability and validity of constructs.

Construct	Items	Standardized loadings	Cronbach’s α	CR	AVE
AI anxiety (AIA)	AIA1	0.82	0.89	0.88	0.65
	AIA2	0.79			
	AIA3	0.85			
	AIA4	0.76			
Job burnout (JB)	JB1	0.88	0.91	0.92	0.70
	JB2	0.85			
	JB3	0.81			
	JB4	0.84			
	JB5	0.80			
Creative self-efficacy (CSE)	CSE1	0.81	0.87	0.88	0.68
	CSE2	0.86			
	CSE3	0.79			
Innovative intentions (INI)	INI1	0.83	0.90	0.91	0.72
	INI2	0.89			
	INI3	0.80			
	INI4	0.85			
Perceived Organizational Support (POS)	POS1	0.80	0.88	0.89	0.67
	POS2	0.84			
	POS3	0.78			
	POS4	0.82			

Model fit indices: χ^2^/df = 2.14; CFI = 0.95; TLI = 0.94; RMSEA = 0.05; SRMR = 0.04. CR, Composite Reliability; AVE, Average Variance Extracted. All loadings are significant at *p* < 0.001.

While Harman’s single-factor test provided an initial assessment, we acknowledge its widely recognized limitations as a standalone test for detecting CMB. To enhance methodological rigor, we additionally conducted an unmeasured latent method construct (ULMC) analysis using the Confirmatory Factor Analysis (CFA) approach (Podsakoff et al., 2003). A common latent factor (CLF) was introduced into the measurement model, connected to all observed items. The results indicated that the addition of the CLF did not significantly improve the model fit, and the variance explained by the latent method factor was only 14.2%, which is well below the 25% median threshold reported in behavioral research. Furthermore, all item loadings on their theoretical constructs remained highly significant even after controlling for the CLF. As a further diagnostic check, we tested whether the pattern of correlations among study variables was consistent with artifactual inflation attributable to common method variance. Specifically, we examined whether the observed correlations exceeded the upper bounds plausible under method-only covariance structures, following the logic outlined by Richardson et al. (2009). The magnitude of the key structural paths–particularly the AI Anxiety → Burnout relationship (*r* = 0.45) and the Burnout → CSE relationship (*r* = −0.50)–falls within the range documented in multi-source studies employing similar constructs, suggesting that common method inflation, if present, is unlikely to account for the substantive pattern of findings. It should be noted, however, that the most rigorous approach to eliminating common method bias–namely, a time-lagged design in which the independent and dependent variables are collected at temporally separated measurement occasions–was not feasible within the constraints of the present study, and is recommended as a methodological priority for future replication. Therefore, statistical evidence, combined with our rigorous procedural remedies, indicates that CMB does not substantially confound the interpretations of our results.

### 5.2 Descriptive statistics and correlations

Table 2 presents the means, standard deviations, and bivariate correlations among the study variables. As anticipated, AI anxiety was positively correlated with job burnout (*r* = 0.45, *p* < 0.001) and negatively correlated with creative self-efficacy (*r* = −0.32, *p* < 0.001) and innovative intentions (*r* = −0.28, *p* < 0.001). Job burnout was negatively associated with creative self-efficacy (*r* = −0.50, *p* < 0.001). Creative self-efficacy showed a strong positive correlation with innovative intentions (*r* = 0.62, *p* < 0.001). These initial correlations provide preliminary support for the hypothesized relationships.

**TABLE 2:** Means, standard deviations, and correlations.

Variable	Mean	SD	1	2	3	4	5
AI anxiety	3.45	0.82	**(0.80)**				
Job burnout	3.10	0.95	0.45**	**(0.83)**			
Creative self-efficacy	3.65	0.78	−0.32**	−0.50**	**(0.82)**		
Innovative intentions	3.72	0.85	−0.28**	−0.48**	0.62**	**(0.85)**	
Perceived Organizational Support	3.30	0.90	−0.20**	−0.35**	0.40**	0.45**	**(0.81)**

*N* = 528.

**p < 0.01. Values in bold diagonal parentheses are the square roots of AVE.

### 5.3 Hypothesis testing: total, direct, and serial mediation effects

We utilized the PROCESS macro (Model 6) with 5,000 bootstrap resamples to test the hypothesized relationships. Before examining the specific mediation pathways, we first assessed the total effect of AI anxiety on innovative intentions without the presence of mediators. The results indicated a significant negative total effect (β = −0.35, SE = 0.05, *p* < 0.001, 95% CI = −0.45, −0.25). This confirms that, overall, higher levels of AI anxiety are associated with reduced intentions to innovate, providing the necessary statistical prerequisite for mediation analysis.

Next, we decomposed this total effect into direct and indirect effects. The results are summarized in Table 3.

**TABLE 3:** Summary of mediation analysis (PROCESS Model 6).

Effect	Path	Coeff. (β)	SE	95% LLCI	95% ULCI	Result
Direct effects	AIA → Job burnout (H1)	0.42***	0.04	0.34	0.50	Supported
	Job burnout → CSE	−0.45***	0.05	−0.55	−0.35	Supported
	CSE → innovative intentions	0.55***	0.04	0.47	0.63	Supported
	AIA → innovative intentions (direct)	−0.08ns	0.04	−0.16	0.00	Non-sig
Indirect effects	Total indirect effect	−0.19	0.03	−0.25	−0.13	Significant
	Ind1: AIA → JB → INI	−0.08	0.02	−0.12	−0.04	Significant
	Ind2: AIA → CSE → INI	−0.05	0.02	−0.09	−0.02	Significant
	Ind3: AIA → JB → CSE → INI (H2)	−0.06	0.01	−0.09	−0.03	Supported

***p < 0.001; AIA, AI anxiety; JB, Job burnout; CSE, creative self-efficacy; INI, innovative intentions. Bootstrap sample size = 5,000.

First, regarding the direct path within the full model, AI anxiety significantly and positively predicted job burnout (β = 0.42, SE = 0.04, *p* < 0.001), supporting Hypothesis 1. However, the direct effect of AI anxiety on innovative intentions became non-significant (β = −0.08, *p* > 0.05) after including the mediators, suggesting a complete mediation.

Second, the serial mediation analysis revealed three specific indirect paths linking AI anxiety to innovative intentions:

Indirect Effect 1 (AI Anxiety → Burnout → Intentions): the indirect effect was significant (β = −0.08, 95% CI = −0.12, −0.04), indicating that burnout simply mediates the relationship. Indirect Effect 2 (AI Anxiety → CSE → Intentions): the indirect effect was significant (β = −0.05, 95% CI = −0.09, −0.02). Indirect Effect 3 (Serial Mediation: AI Anxiety → Burnout → CSE → Intentions): crucially, the specific serial indirect effect was significant (β = −0.06, SE = 0.01, 95% CI = −0.09, −0.03). The 95% confidence interval did not contain zero. This confirms that AI anxiety reduces innovative intentions by first increasing job burnout, which subsequently diminishes creative self-efficacy, thereby supporting Hypothesis 2.

Collectively, the total indirect effect (sum of the three indirect paths) accounted for 75% of the total effect, highlighting that the effect of AI anxiety is predominantly transmitted through these psychological mechanisms rather than directly.

### 5.4 Moderated mediation analysis

**FIGURE 2:**
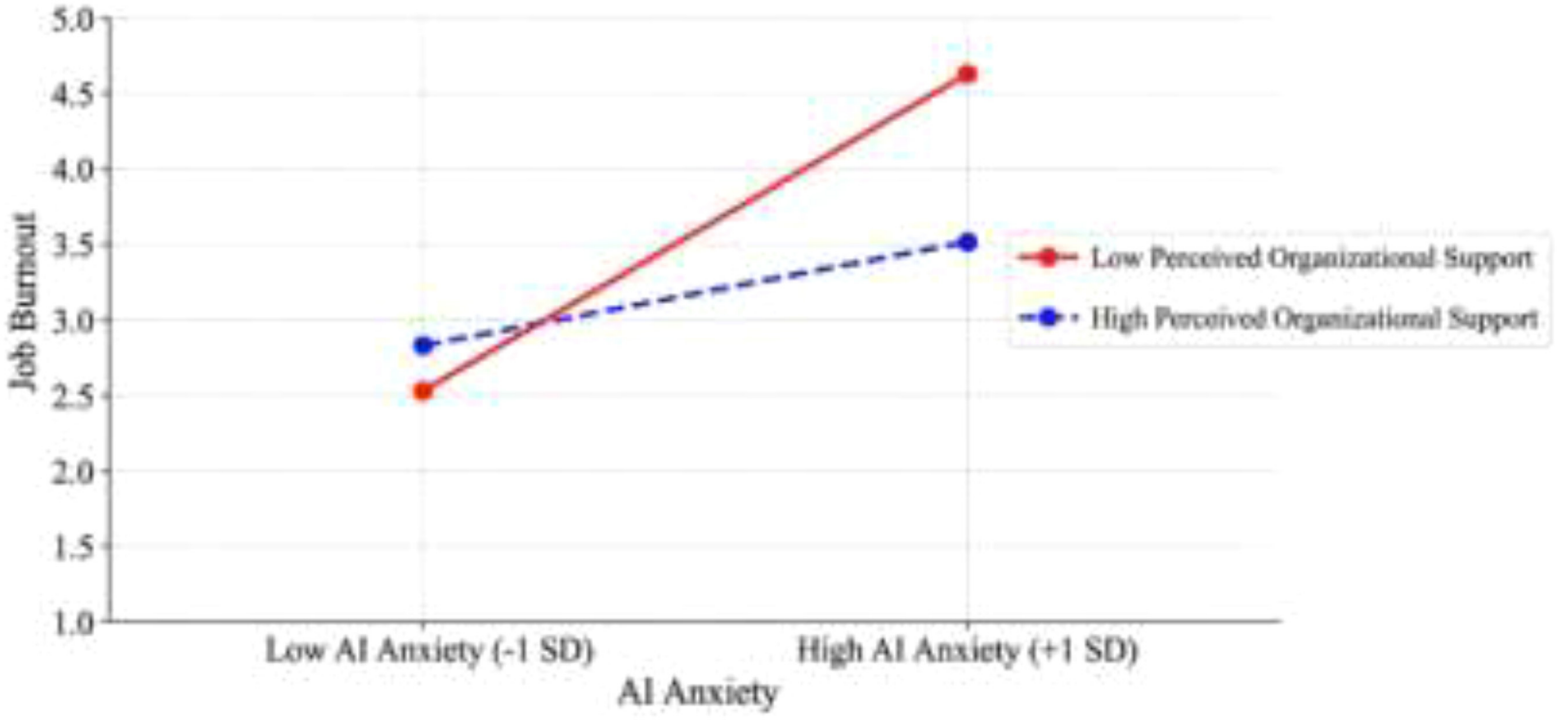
Simple slope analysis.

To test the moderating role of Perceived Organizational Support (POS) on the relationship between AI anxiety and job burnout (first stage), we employed PROCESS Model 83. The interaction term (AI Anxiety × POS) significantly predicted job burnout (β = −0.15, SE = 0.03, *p* < 0.01). The negative coefficient indicates that POS buffers the positive effect of anxiety on burnout. To interpret this interaction, we conducted a simple slope analysis (see Figure 2). The results showed that the positive relationship between AI anxiety and burnout was stronger for teachers with low POS (−1 SD) (simple slope = 0.55, *p* < 0.001) compared to those with high POS (+1 SD) (simple slope = 0.25, *p* < 0.01). Thus, Hypothesis 3 was supported.

Finally, we examined the index of moderated mediation to test Hypothesis 4. The index for the serial mediation path (AI Anxiety → Burnout → CSE → Intentions) was significant (Index = 0.02, SE = 0.01, 95% CI = 0.005, 0.040). Specifically, the indirect negative effect of AI anxiety on innovative intentions via the serial chain was stronger when POS was low (indirect effect = −0.08) and weaker when POS was high (indirect effect = −0.03). This confirms that organizational support attenuates the detrimental chain reaction initiated by AI anxiety (see Table 4).

**TABLE 4:** Moderated mediation results (index of moderated mediation).

Effect analysis	Moderator level	Effect	Boot SE	95% LLCI	95% ULCI
Conditional indirect effect					
	Low (−1 SD)	−0.08	0.02	−0.12	−0.05
	Mean	−0.05	0.01	−0.08	−0.03
	High (+1 SD)	−0.03	0.01	−0.05	−0.01
Index of moderated mediation					
(Test of H4)		0.02	0.01	0.005	0.040

## 6 Discussion

The present study set out to explain why the proliferation of generative AI in education does not automatically translate into teacher innovation, and may in fact undermine it. Drawing on COR theory and SCT, we proposed and empirically validated a moderated serial mediation model in which AI anxiety impairs innovative intentions by sequentially depleting emotional resources (burnout) and eroding cognitive agency (creative self-efficacy), with Perceived Organizational Support moderating the initial stress-depletion link. The findings support all four hypotheses and clarify the specific process through which technological anxiety becomes behavioral withdrawal.

### 6.1 Theoretical implications

This study advances the literature on educational technology and organizational psychology in three significant ways.

First, it challenges the “pro-innovation bias” in EdTech research by elucidating the “dark side” of AI integration. Prevailing literature often assumes a direct, positive correlation between technology access and pedagogical innovation (the Technology Acceptance Model). However, our findings corroborate the COR perspective that the introduction of disruptive technology is often initially perceived not as an opportunity, but as a threat to resource stability (Skaalvik and Skaalvik, 2017). We found that AI anxiety is a significant positive predictor of job burnout (H1). This supports the notion that the continuous cognitive appraisal of AI as a complex, opaque, and threatening force requires a sustained investment of “energetic resources” (Hobfoll, 1989). This finding constitutes a theoretical corrective to the TAM-dominated literature: it demonstrates that AI exposure is processed, at least initially, through a threat-appraisal lens rather than a utility-appraisal lens, and that emotional resource depletion precedes–and may preclude–any meaningful engagement with the question of whether the technology is useful. In practical terms, this implies that adoption barriers are not primarily cognitive (insufficient knowledge of AI features) but affective (insufficient psychological safety in engaging with AI), a distinction with significant consequences for how professional development programs are designed.

Second, this study makes a cohesive theoretical contribution by explicating a process-based explanation (not just variable relationships) for how stress translates into inaction. While the synthesis of COR and SCT provides the macro-framework, the true theoretical innovation lies in mapping the precise “depletion → cognition → behavior” mechanism. Previous research often treats emotional depletion and cognitive efficacy in isolation. To be precise, this sequential dependency constitutes the core theoretical contribution distinguishing the present model from prior mediation-based accounts. Studies such as Gong et al. (2009) and Urban and Urban (2025) position creative self-efficacy as an independent mediator between leadership or environmental antecedents and creative output, without routing it through a prior depletion state. Similarly, Salmela-Aro et al. (2019) document the burnout-performance link without examining whether this effect operates through the specific channel of eroded efficacy beliefs. The present study establishes that the two mechanisms are not merely additive but constitute a temporally ordered chain: anxiety drains emotional resources (COR), and it is precisely from the experienced state of depletion that teachers construct their appraisal of cognitive insufficiency (SCT). Exhaustion, in this account, does not directly suppress innovative behavior; it does so by providing the somatic evidence upon which a disabling self-assessment is built.

Third, the moderating role of Perceived Organizational Support (H3 & H4) highlights the contextual contingency of the stress process. Our findings reveal that POS functions as a vital “resource caravan” that buffers the pathogenic effects of AI anxiety. Specifically, high levels of organizational support attenuate the link between anxiety and burnout, effectively blocking the negative spiral at its inception. This lends empirical support to the buffering hypothesis within the COR framework. It implies that external institutional resources (emotional backing, technical safety nets) can compensate for the internal resource loss triggered by technological stress. When teachers feel “backed” by their organization, the cognitive appraisal of AI shifts from a solitary threat to a manageable challenge, thereby preserving the emotional and cognitive resources necessary for innovation.

### 6.2 Practical implications

For educational administrators and policymakers, these findings translate into three actionable strategies for fostering innovation in the AI era.

Address the Psychological “Root,” Not Just the Technical “Branch”: School leaders must recognize that resistance to AI often stems from fear and resource depletion rather than stubbornness or lack of skill. Therefore, professional development should pivot from purely functional training (how to use the tool) to psychological inoculation. Interventions such as “AI decomplexification” workshops can help demystify the technology, framing it explicitly as an “augmented intelligence” tool designed to assist rather than replace teachers. By reducing the opacity of the technology, schools can lower the initial anxiety threshold. For example, a professional development cycle might begin not with instruction on AI functionality but with a structured reflection exercise in which teachers identify one routine task–such as generating differentiated reading questions–that they find cognitively burdensome, followed by a guided demonstration of how an AI tool can handle that task. This “burden-first, tool-second” sequencing reframes AI as responsive to a teacher-defined need rather than as an externally imposed competency requirement, directly targeting the professional autonomy threat identified in the anxiety literature.

Implement “Resource-Neutral” Integration Strategies: To prevent the burnout identified in our model, administrators must ensure that the introduction of AI does not simply add to teachers’ existing cognitive load. We recommend a “resource-neutral” or “resource-additive” approach: for every new AI-based task introduced, an obsolete administrative burden should be removed. For example, if teachers are expected to use AI for personalized lesson planning, schools should simultaneously automate routine grading or attendance tasks. This conservation strategy ensures that teachers retain the “energetic resources” required for high-level creative thinking. A concrete implementation example: if teachers are required to incorporate AI-generated formative assessments into their practice, the school should simultaneously suspend the requirement to submit weekly written lesson reflections, redistributing rather than inflating the total cognitive burden. Resource budgeting of this kind is analogous to the workload-neutral change management protocols used in organizational redesign (cf. Hobfoll, 1989) and operationalizes COR theory’s prescription that resource investment must be offset by resource conservation.

Cultivate a Psychologically Safe “Innovation Sandbox”: The buffering effect of POS suggests that the school environment acts as a protective shield. Leaders should actively cultivate a climate of psychological safety–an “innovation sandbox”–where experimentation with AI is encouraged and failure is framed as learning rather than incompetence (Edmondson, 1999). When teachers know that the organization values their well-being (high POS) and will not penalize them for the initial inefficiencies associated with learning new tools, their creative self-efficacy is protected, and their willingness to innovate persists even in the face of anxiety. Structurally, this might take the form of a designated “AI experimentation period”–for instance, one lesson per fortnight in which teachers are explicitly released from outcome accountability and encouraged to trial AI-assisted instructional designs. The key design feature is institutional endorsement of failure as informative rather than evaluative: Edmondson’s (1999) research on team psychological safety demonstrates that perceived permission to fail is the most reliable predictor of the exploratory behavior that innovation requires. Schools in which administrators publicly acknowledge their own uncertainties about AI, and treat teacher experimentation as organizational learning, are likely to produce the high-POS climate that our moderating findings suggest is protective.

### 6.3 Limitations and future directions

While this study offers valuable insights, several limitations warrant consideration and suggest avenues for future research.

First, the cross-sectional design of this study fundamentally precludes the establishment of definitive causal inferences. Although we framed our relationships in a directional manner based on robust theoretical antecedents (COR and SCT), the data only confirm statistical associations rather than true causality. We must explicitly acknowledge the potential for alternative explanations, including reverse causality. For instance, it is highly plausible that teachers who inherently possess lower creative self-efficacy or are already experiencing burnout may perceive new AI technologies as more threatening, thereby reporting higher levels of AI anxiety. Furthermore, the relationships among these variables are likely dynamic and reciprocal over time, potentially forming a continuous feedback loop where anxiety and withdrawal reinforce each other. Future research must expand on these limitations by employing longitudinal cross-lagged panel models or experience-sampling methods (diary studies) to capture the dynamic, day-to-day unfolding of these psychological states and to rigorously disentangle the causal directionality of the “depletion-cognition-behavior” mechanism.

Second, despite procedural remedies and statistical checks (Harman’s single-factor test) indicating that common method bias was not a pervasive issue, the reliance on self-reported measures remains an inherent limitation. Self-assessments of innovative intentions may be influenced by social desirability bias or subjective overestimation, potentially differing from actual classroom behaviors. Future studies could benefit from multi-source data, incorporating peer ratings, supervisor evaluations, or objective technology usage logs to enhance ecological validity. Additionally, a time-lagged survey design–collecting antecedent variables (AI anxiety, POS) at Time 1 and outcome variables (burnout, CSE, innovative intentions) at Time 2, separated by an interval of 4–8 weeks–would provide a more defensible basis for causal inference while simultaneously addressing the most fundamental limitation of single-wave, single-source data. Designs of this type would permit direct assessment of whether the observed correlations reflect prospective prediction rather than concurrent association.

Third, the sample’s cultural context may limit generalizability. Our data were drawn from China, a context often characterized by high power distance and collectivism. In such cultures, organizational support may carry different weight than in individualistic contexts. Cross-cultural comparative studies would be valuable to test the universality of the proposed model. For instance, Does POS buffer anxiety as effectively in educational systems with higher teacher autonomy? Exploring these boundary conditions will be crucial for developing a globally applicable theory of teacher adaptation to AI.

## 7 Conclusion

This study moves beyond the prevalent technological determinism to unveil the hidden psychological costs of digital transformation in education. By delineating the “Exhaustion-Doubt-Withdrawal” pathway, we highlight a critical paradox: the very pressure to innovate with AI may inadvertently suppress the human capacity to do so. Our findings reveal that teacher innovation is not merely a function of technical skill or administrative mandate, but is deeply rooted in the preservation of emotional and cognitive resources.

Broader than the specific relationships between anxiety and burnout, this research underscores the pivotal role of the affective dimension in the future of human-AI collaboration. As education systems race toward digitalization, our results serve as a cautionary tale: neglecting the “human factor”–specifically, the psychological vulnerability of educators–can lead to a hollow transformation where technology is present, but meaningful human engagement is absent. The significant moderating role of organizational support further suggests that the “AI divide” may not be just about access to technology, but about access to psychological safety and institutional backing.

Ultimately, this study advocates for a paradigm shift toward “Sustainable Innovation.” Innovation that comes at the cost of teacher well-being is neither ethical nor durable. True educational advancement requires an ecosystem where AI is positioned not as a competitor that induces anxiety, but as a partner that is introduced with empathy. Future educational policies must therefore prioritize “resource conservation” alongside “technological acceleration,” ensuring that teachers are empowered, rather than exhausted, by the intelligent age.

## Data Availability

The data analyzed in this study is subject to the following licenses/restrictions: the data analyzed in this study are not publicly available due to privacy and ethical considerations related to the human participants. Access to the data may be granted to qualified researchers upon reasonable request and subject to approval by the corresponding author. Requests to access these datasets should be directed to TD, qfnudtq@qfnu.edu.cn.
